# Modelling and the Nation: Institutionalising Climate Prediction in the UK, 1988–92

**DOI:** 10.1007/s11024-016-9302-0

**Published:** 2016-07-04

**Authors:** Martin Mahony, Mike Hulme

**Affiliations:** 1 School of Geography, University of Nottingham, University Park, Nottingham, NG7 2RD UK; 2 Department of Geography, King’s College London, London, UK

**Keywords:** Climate change, Models, Science and policy, Environmental politics, Civic epistemology

## Abstract

How climate models came to gain and exercise epistemic authority has been a key concern of recent climate change historiography. Using newly released archival materials and recently conducted interviews with key actors, we reconstruct negotiations between UK climate scientists and policymakers which led to the opening of the Hadley Centre for Climate Prediction and Research in 1990. We historicize earlier arguments about the unique institutional culture of the Hadley Centre, and link this culture to broader characteristics of UK regulatory practice and environmental politics. A product of a particular time and place, the Hadley Centre was shaped not just by scientific ambition, but by a Conservative governmental preference for ‘sound science’ and high evidential standards in environmental policymaking. Civil servants sought a prediction programme which would appeal to such sensibilities, with transient and regional climate simulation techniques seemingly offering both scientific prestige and persuasive power. Beyond the national level, we also offer new insights into the early role of the Intergovernmental Panel on Climate Change and an evolving international political context in the shaping of scientific practices and institutions.

## Introduction

Complex scientific models of the climate system have occupied a prominent place in the politics of climate change since the 1970s. In particular, general circulation models (GCMs) have offered quantitative estimates and qualitative visions of putative futures, which have found their way into broader cultural narratives of climate change. These narratives oscillate between the apparent certitude of impending crisis and the hazy, disarming uncertainty of innumerable possible outcomes of environmental and social change. Epistemologically speaking, climate models pose challenges to the assumed independence of theory, experiment and observation (Dowling 1999; Sismondo 2008). Culturally, they unsettle accepted boundaries between fact and value, science and politics (Jasanoff and Wynne 1998; Weingart 1999; Walsh 2009; Hulme 2011). Politically, models render environmental change as a problem which can only be suitably addressed at the global scale (Ashley 1983; Demeritt 2001; Miller 2004; Oels 2005; Dahan 2010; Hulme 2010). Long-term model projections of the ‘earth system’, replete with a fecundity of interrelated, human/nonhuman sub-systems, offer a challenging totality which seemingly must be carefully managed within prescribed limits and boundaries (e.g. Rockström et al. 2009).

In this paper we seek to explore the relationship between climate modelling and climate change policy. More specifically, we seek to historicize the close relationship between climate modelling and climate politics which has been observed by many scholars (e.g. Shackley 2001; Demeritt 2001; Miller 2004; Edwards 2010). This observation of a close relationship has been made from a number of standpoints: the natural proximity of facts and values (Winsberg 2010; Intemann 2015), the intertwining of knowledge and power in the ‘governmentalisation’ of climate change (Oels 2005), the technological ‘enframing’ of human action (Hamilton 2015), or the co-production of global knowledge and nascent global political orders (Miller 2004, 2009). Climate models have been understood as key actors at the ‘science-policy interface’, mediating between the social worlds of science and politics, and fundamentally shaping understanding of, and even responses to, climate change. While we concur with many of these analyses, we also think it important to attend closely to the social constitution of the science-policy interface, in order to explain exactly how it is that this modelling-politics nexus has attained the shape that it has today. Such an approach is not without precedent (see below), but we contend that more fine-grained studies of the spaces where science and politics meet are needed to make sense of the imbrications of knowledge and power, facts and values, in the social world of climate change discourse.

Our study focuses on the institutionalisation of climate modelling and prediction in the UK in the late 1980s. The period between 1985 and 1990 was a formative one for the science and politics of climate change (Agrawala 1998). The Villach Conference of 1985 produced influential scientific statements that ‘significant climate change’ was ‘highly probable’, and recommended states start consideration of a global climate convention (WMO 1986). 1988 saw the formation of the Intergovernmental Panel on Climate Change (IPCC) and the World Conference on the Changing Atmosphere in Toronto, the latter recommending cuts to global CO_2_ emissions of 20% by 2005 (Bodansky 1995). On an unusually hot day in June 1988, NASA’s Jim Hansen gave testimony to the US Senate, including observational evidence that the earth was now warmer than at any other time in the instrumental record. Hansen famously argued that “the greenhouse effect has been detected, and it is changing our climate now”. Responding to the prevailing weather conditions and appealing to the thermal discomfort of the sweltering hearing room, Hansen told the committee that “our simulations indicate that the greenhouse effect is already large enough to begin to effect the probability of extreme events such as summer heat waves” (Hansen 1988). Hansen’s testimony was one among a number of high-profile media events at the time which pushed climate change into the public sphere, bringing with it increasingly confident knowledge claims about future climate which rested on climate models. Hansen’s testimony neatly illustrated the new epistemic power of climate models to offer convincing visions of the future, and to potentially transform understandings of the climatic present.

It is nonetheless important to attend to the variegated geographies of this ‘epistemic power’ (Mahony and Hulme 2012: 208). Three-dimensional climate modelling is a specialised activity and GCMs are only operated at a few institutional locations worldwide where the requisite expertise combines with high levels of financial support (Edwards 2010). These modelling centres exhibit different institutional cultures, expressed, for example, in different management structures, research priorities, academic networks, working styles and links to policymakers. These differences have been elucidated through sociological and ethnographic work, most notably by Shackley (2001) and Krueck and Borchers (1999), and through studies of institutional histories (e.g. Howe 2014). Our historiographical contention is that studying moments of institutional formation can yield important insights into how the kinds of scientific cultures or ‘epistemic lifestyles’ described by Shackley came about, thus offering explanatory resources for the varying styles of climate modelling which are discernible to this day.

Furthermore, following Jasanoff (2004), institutions can be understood as key sites for the co-production of knowledge and social order, where new knowledge-making practices emerge alongside new ways of thinking about and ordering social relations. “As stable repositories of knowledge and power, institutions offer ready-made instruments for putting things in their places at times of uncertainty and disorder”. In institutions such as scientific laboratories or judicial systems, “societies have access to tried-and-true repertoires of problem-solving, including preferred forms of expertise, processes of inquiry, methods of securing credibility, and mechanisms for airing and managing dissent” (ibid: 39–40). In the context of climate change, much analytical attention has been directed at institutional innovation, in studies of how societies have developed new institutional structures to deal with the ‘uncertainty and disorder’ of a changing climate (e.g. Shackley and Wynne 1995; Nolin 1999; Miller 2004). Likewise, histories of climate science have focused on an internal story of discovery and institution-building, arguably without sufficient regard for cross-sectoral continuity in institutionalised ways of producing and authorising knowledge (e.g. Weart 2010; Edwards 2010).

Our case in this paper is the establishment of the Hadley Centre for Climate Prediction and Research at the UK Met Office. The Hadley Centre was opened in 1990, at the height of the early political interest in climate change which culminated in the adoption of the United Nations Framework Convention on Climate Change (UNFCCC) in 1992. Making use of newly available archives^1^ as well as recently conducted interviews with key actors in science and government, we reconstruct the deliberations between scientists, research managers and policymakers which led to the establishment of the UK’s first and only dedicated climate change prediction programme.^2^ The case points to the importance of political context in both creating the conditions for new kinds of knowledge production, and in shaping the kinds of knowledge which have been pursued at the science-policy interface. We argue that the period between 1988 and 1992 saw a concerted re-patterning of the horizontal relationships between atmospheric science and government, and of the vertical relationships between national and international modes of climatic knowledge-making, in a fashion which reflects changes observable in other scientific fields and policy domains, both at the time and since (Miller 2009). Concerning the horizontal relationships, we argue that the object of climate change, and the technology of climate models, effected new relationships between scientists and political actors, extending from civil servants to the UK Prime Minister at the time, Margaret Thatcher. In the vertical dimension, we argue that the establishment of the IPCC had important effects on the conduct of climate science – an effect which has been appreciably under-studied in contemporary science studies. But we also argue that these transformations can be understood within a wider context of institutionalised forms of knowledge-making in the UK. It is a story of radical change, but we wish to emphasise how the shape of that change was importantly shaped by what had gone before.

## The New Politics of Climate in the UK

Prior to the 1940s, the climatological section of the Meteorological Office was an unassuming division of data-gatherers and book-keepers, dependent on a network of around 5,000 amateur weather observers. Following the Second World War, climatological information began to attract the interest of the utilities sector, particularly energy suppliers, motivated in part by climatological extremes such as the severe winter of 1946/7 (Hall 2012). However, with climatological work split between forecast services and research divisions, climatology in the Met Office^3^ was essentially defined as that which wasn’t synoptic meteorology (Hall 2015a). It was arguably Hubert H. Lamb and Gordon Manley who developed new links in the UK between the record-keeping style of climatology and scientists concerned with the dynamics of the atmosphere, introducing ideas of climatic change at large scales. In the 1960s, as interest in the general circulation and the possibilities of large scale climatic change and even control took hold, John Mason (Director-General of the Met Office 1965-83) placed new emphasis on computational facilities both for purposes of numerical weather prediction and for the newly re-organised Dynamical Climatology and Synoptic Climatology research groups. The immediate post-war period had seen the Met Office consolidate its near-monopoly position as a national weather information provider, as the technical advances of World War II were paired with a new, public- and business-facing ethos of service provision. Although commercial logics grew in prominence internally, the Met Office never encountered the kind of private competition which the US Weather Bureau, for instance, was faced with. Mason capitalised on the Office’s new public profile and resources when he took office, with one of his first acts being the operationalisation of numerical weather prediction (Hall 2015b). Generous support from the Ministry of Defence (MoD) bolstered this computational turn, and also meant the Met Office could resist being pulled into the orbit of newly established government research funders, such as the Natural Environment Research Council (NERC). International links, promoted by Mason through organisations like GARP (the Global Atmospheric Research Program, 1967–1982) under the auspices of the World Meteorological Organisation (WMO), contributed to the acceleration in modelling capacities as a Met Office GCM was built up (Walker 2012; Hall 2015b).

The Met Office was aware of and involved in the earliest international scientific debates about global climate change in the 1960s, but it wasn’t until the late 1970s that climate change as a potential policy issue had its first encounter with the UK Government. As Agar (2015) has recently shown, the Conservative government’s first encounter with climate change was far from straightforward. The Interdepartmental Group on Climatology (IGC), convened in October 1978 under the chairmanship of economist Kenneth Berrill, was commissioned by the Labour government in 1979 to write a review of the science of climate change. By the time of its publication, the Conservatives were in power and the new administration was clearly keen to distance itself from the report, with a steer apparently coming from the very top. The report concluded that climatic fluctuations over the next century were unlikely to be smaller than those of the preceding century, that the influence of fossil fuel burning was the most serious cause for concern, and that more research was needed. After a delay, the report was “grudgingly” put out by the government, amid worries about its lack of a real message. Accepting that the report might nonetheless help display to pressure groups a genuine concern for long-term environmental issues on the part of the government, the report was published in February 1980, receiving a fairly ‘cool’ response both in the media, and from Margaret Thatcher herself (Agar 2015).

Fast-forward to the 1990s, and a very different picture of the horizontal alignment of climate science and politics emerges. This picture has been mostly clearly painted by Shackley (2001), drawing on ethnographic observations of climate modelling practice, his rendering of ‘epistemic lifestyles’ and institutional cultures made sharper through his comparative methodology. While the National Center for Atmospheric Research (NCAR) in Colorado displayed a largely non-hierarchical structure, a focus on ‘pure’ research and relative intellectual freedom for researchers (see also Howe 2014), the Hadley Centre for Climate Prediction and Research at the Met Office exhibited what Shackley called a ‘hybrid’ modelling style. ‘Climate seers’ dominated the modelling activities – i.e. those interested in exploring and experimenting with the modelled climate system and its changes, as opposed to ‘model constructors’ who are more interested in the construction of complex models as an end in itself.^4^ The latter style is perhaps more akin to the culture observed by Krueck and Borchers in the Hamburg network of climate modellers, at the centre of which sat the Max Planck Institute for Meteorology and the German Climate Computing Centre (DKRZ); a culture which they compared to a relatively “more focused and conservative” style of research at the Hadley Centre where the emphasis was on the delivery of “sound, useable science” (Krueck and Borchers 1999: 117). Crucially, the Hadley Centre had a very different relationship to policymakers than any of its American or German rivals: “the policy-influenced objectives and priorities of the research organization, as defined by its leadership, take preference over other…motivations and styles” (Shackley 2001: 128). In Hamburg, modelling was largely “aloof from politics” (Krueck and Borchers 1999: 123). At the Hadley Centre, through negotiations with a small “coterie of policymakers”, money was allocated to the organization as a whole and research priorities set (Shackley 2001: 128; see also Shackley and Wynne 1995, 1996).

Our interest in this paper is in how the relationship between climate science and the UK government changed so dramatically in the intervening years between Thatcher’s early coolness and the formation of a coterie of prediction-hungry policymakers. In the next section, we describe how ideas for a new ‘National Climate Centre’ began to take shape in the late 1980s.

## A ‘National Climate Centre’

From the very start of recorded discussions of what was initially referred to as a National Climate Centre, it is clear that the establishment of the IPCC by the WMO and United Nations Environment Programme (UNEP) was influential in key actors’ thinking about the epistemic demands posed by an emerging politics of climate. In an oral history given to the British Library, Sir John Houghton (Director General/Chief Executive of the Met Office, 1983–91) responded to a question about the formation of the Hadley Centre by referring immediately to the new international body: “the IPCC was key to that” (Houghton and Merchant 2011: 180). Houghton attended the first IPCC meeting in Geneva in November 1988 along with David Fisk, the Department of the Environment’s (DoE) Chief Scientist. With Fisk’s support, Houghton accepted the chairmanship of IPCC’s Working Group I and DoE pledged resources to support the scientific and clerical work which would be required to conduct the assessment. Geoff Jenkins, a Met Office scientist who had been on secondment at DoE until late 1988, was given the task of heading up this early version of what would later come to be called an IPCC Technical Support Unit. But not all of the Met Office’s directors were convinced that climate change came under the remit of a state meteorological service:

There were people who were not particularly interested in the whole climate issue I guess. And they felt the Met Office had a big enough job to forecast the weather…so to get sidestepped into doing things that weren’t directly our job was not what they wanted to do. Because, as I say, it clearly wasn’t in our remit to do it. I couldn’t have got the sort of millions to spend on that sort of thing from MoD (Sir John Houghton, interview).

Despite these reservations, Houghton moved quickly to start gathering the resources required for a new climate change centre which could act as a focal point for the UK’s input into the IPCC process: “we were getting on with it” (ibid). Four days after the Geneva meeting where the idea of the DoE supporting a new research programme was seemingly born, Houghton wrote to Fisk with a proposed ‘National Plan for Climate Change’, calling for an expansion of both observational studies and predictive modelling. The latter, however, was the clear priority and was to be the focus of any new resources. While paleoclimatology – the reconstruction of past climates using non-instrumental sources such as tree rings – was considered “valuable in relation to the assessment of future climate change”, Houghton suggested that it “probably needs little or no extra stimulation”. The success of earlier model simulations at the Met Office of climate (e.g. Gilchrist et al. 1973), and of equilibrium climate change (e.g. Mitchell 1983; see also Folland et al. 2004), meant that the modelling programme should be “accelerated” in order to inform emerging policy debates.^5^ Yet the salience of these policy debates was not something which was expected only to emerge *after* a firm scientific picture had first been produced. Rather, the political context of climate change was already taking shape.

On 27th September 1988, Margaret Thatcher had given a speech to the Royal Society’s annual dinner in which she proffered her new concern about global warming and environmental change. After extolling the virtues of “keenly competitive” researchers and their centrality to “national prosperity and international standing”, Thatcher expressed unease about the environmental impacts of the massive changes wrought by industrialisation, specifically in terms of population growth, agriculture and the use of fossil fuels. “It is possible”, she famously suggested, “that…we have unwittingly begun a massive experiment with the system of this planet itself”. With the “health of the economy and the health of our environment…totally dependent on one another”, environmental policy measures could be rationally justified, especially with the help of Britain’s world-leading climate scientists. Nonetheless, Thatcher hinted at high evidential standards for the scientific knowledge upon which any climate policy would be based: “We must ensure that what we do is founded on good science to establish cause and effect” (Thatcher 1988).

Scholars of regulatory and advisory science have noted a preference in British environmental policy for high evidential standards. Jasanoff in particular has observed a British policy culture and ‘civic epistemology’ which “shares with British science a pragmatic, empirical orientation, producing scepticism about claims that appear to go beyond the observable facts of nature or society” (2005: 263). In early environmental policy debates, “regulatory policy was based only on what could be known with near absolute certainty” (ibid: 264; see also Brickman et al. 1985; Jasanoff 1991). One of our interview respondents, reflecting on his time as a scientific advisor at DoE, linked this trait explicitly to Conservative attitudes to environmental issues, explaining that government figures would “need to be convinced” that any policy measures were

being done on a good, sound scientific basis, because their supra-policy was that they would do anything as long as there was good scientific evidence for it. And then they’d have very high barriers for what constituted evidence (DoE science advisor, interview).

For another DoE official (interview), this emphasis on ‘sound science’ as a basis for policy “became explicit during the Conservative administration”, and was expressed particularly clearly in deliberations concerning ozone depletion and acid rain, where Britain’s reputation as the laggardly ‘Dirty Man of Europe’ was enhanced by a reluctance to sign acid rain treaties without first waiting for scientific clarity over causal mechanisms, where others operationalised the precautionary principle (see Rose 1990):

In fact it was Margaret Thatcher who said almost precisely that: ‘how many salmon will shutting down Drax [Britain’s largest coal-fired power station] save?’ We don’t know. Now, with work, we can get towards an answer (DoE official, interview).

Convinced of the need for action on acid rain in the early 1980s, DoE officials turned to advanced computer modelling as a means of offering ministers clearer pictures of transnational chemical causation, as the concept of ‘critical loads’ gained traction as a means of precisely defining how much pollutant a specific, local ecosystem could absorb before damage occurred (Nilsson 1988).^6^ While Jasanoff detects in British civic epistemology and environmental politics “an empiricist distrust of untested and overextended models”, even a “residual scepticism toward *all* model-based predictions” (Jasanoff 2011: 137, emphasis in original), it seems that by the middle of the 1980s, DoE “were very used to modelling as a basis of prediction”, policymaking and policy enforcement, as departmental experts made use of simulated chemical transport to critique other countries’ reported sulphur dioxide emissions (DoE official, interview). In this period then, we can begin to see how the ‘pragmatic empiricism’ of UK regulatory culture was being reconciled with what we might see as a contrary reliance on models, simulation and prediction. Amid demands for high evidential standards, civil servants and policymakers alike were developing confidence in particular environmental models which could act as quasi-empirical surrogates for deficient data or intractable causal complexity.

A strain of this pragmatic empiricism can be detected in a meeting of British climate scientists convened in response to Thatcher’s Royal Society speech. The meeting on 7th November 1988 was convened under the auspices of the British National Committee of the World Climate Research Programme (WCRP) to “discuss the scientific community’s response to the speech made by the Prime Minister”. John Mason was in the chair and penned the report, which was clearly aimed at the atmospheric sciences’ national paymasters. The assembled members took the view

that a realistic investment in research (as part of planned international programmes) and aimed at narrowing the wide range of current estimates, would be a prudent and small insurance premium relative to the enormous costs that would be involved in attempting to ameliorate the impact of the worst of these predictions.^7^

The ‘wide range of current estimates’ referred mainly to the results of equilibrium experiments with GCMs, whereby the models were run with doubled CO_2_ levels until the climate reached a new stable state. Most of these experiments by 1988 had been conducted at US modelling centres. For the committee, it therefore appeared important that

the UK, acting perhaps on behalf of Europe in this matter should maintain the ability to make independent judgements on such important and controversial issues. Because of our leadership in European meteorology and our reputation for critical and balanced judgements in these matters, the UK could easily take the lead in developing a European stance on these issues, which are bound to attract some wild and conflicting views.^8^

In these words we can hear the characteristic voice of Mason, who had been known in government circles for his scepticism about the potential for long-term or catastrophic climatic changes since the 1970s (see Agar 2015; Mason 1976). A cloud physicist by training and a keen advocate of new computing methods in the face of some intransigence within the Meteorological Office (Martin-Nielsen 2015; Hall 2015b),^9^ Mason had responded to early predictions of anthropogenic climate change in a way which chimes with Jasanoff’s posited culture of pragmatic empiricism. In the late 1970s Mason had recognised the “potential importance and concern” of the topic, advocating for a “sustained research programme” to improve understanding of past and current trends, and their underlying mechanisms (Mason 1976: 473). He had “set out to debunk United States alarmist views” (Mason, quoted in Agar 2015: 15) and, through the establishment of a new committee of scientists and government figures in 1977, to exert “a measure of control”, as Agar puts it, “from a sceptical Met Office point of view” on a topic about which other institutions were starting to establish a strong voice (Agar 2015: 16). Mason’s bid to exert some control, both through committees and by stimulating new modelling work within the Met Office, was clearly a response to outside arguments which, to paraphrase Jasanoff (2005), appeared to contradict accepted and ‘observable’ facts about climate and society (see Mason 1976; for a retrospective account, see Mason 2010).

After a decade of further model development, Mason was still convinced by the need for a “sane and critical voice outside the United States” (quoted in Rose 1990: 299), and the British Committee of WCRP meeting in November 1988 argued for investment in modelling to encourage improvement “to the stage at which their predictions converge and thereby provide more reliable advice to governments”.^10^ These arguments were being made at a time when close links were being developed between the Meteorological Office and DoE. From the mid-1980s, Met Office scientists had been sent on secondments to DoE in order to feed knowledge into other atmospheric policy domains like acid rain and ozone depletion. Although the issue of climate change had not been on the agenda at the start of the secondments programme, the presence of Met Office employees in DoE was significant as international reports like that produced in Villach (WMO 1986) pushed climate up the political agenda, helping smooth the way to new cooperative and financial relationships.

Likewise, a Met Office scientist had been sent to the Natural Environment Research Council (NERC) to help sharpen that body’s focus on the atmospheric sciences, which until that point had received just 0.4% of NERC’s funding.^11^ In 1987, NERC had established UGAMP, the UK Universities Global Atmospheric Modelling Project, centred on the University of Reading. While Met Office modellers had been refining their analyses of the equilibrium effects of increased CO_2_, UGAMP scientists were focusing largely on processes, for example, seeking to better understand the mechanisms of cyclogenesis or the behaviour of the stratosphere. But it is clear that the UGAMP network was never considered as an alternative to the Met Office as a home for a new national modelling centre. UGAMP scientists were focusing purely on atmospheric modelling, rather than the kind of coupled ocean-atmosphere modelling required for long timescale climate prediction, while the wider NERC community had not engaged in the development of a global ocean model (interview, Met Office research manager and former NERC secondee). The Met Office was therefore considered the natural home for the new prediction programme:

if you wanted to be able to have an independent view, and compete with the work that was going on particularly in the USA at this stage, on that type of modelling to address the global warming type issues, then I think the Met Office was the place (ibid).

With its existing observational programmes and modelling capacities strengthened by both Mason and Houghton, and with its historical centrality as a provider of public weather knowledge (Hall 2015b), the Met Office was widely considered to be the natural place where “the nation’s resources should be drawn together to focus effort on modelling the climate system”.^12^ DoE actors, particularly Chief Scientist David Fisk, had been convinced by the Met Office’s early advances, and conversations began about the exact form a new climate centre would take.

### *Shaping the Centre*

The Met Office’s core funding in the late 1980s came from the Ministry of Defence (MoD), but the MoD’s interest in climate did not extend to long-term prediction. Nonetheless pockets of research pertinent to the climate change question existed throughout the Met Office, particularly in the Synoptic and Dynamic Climatology branches, and in the model-building capacities of the numerical weather predictors. Houghton’s ambition was to build on an existing culture of inter-branch collaboration, to gather these fragments together into a new whole which would in turn become a “focus” for other modelling efforts in the UK.^13^ Despite the aforementioned internal objections to the Met Office becoming too involved with climate change, which were soon overcome, Met Office actors saw themselves as the only real option for a new national centre. Yet from a DoE perspective, the Met Office offered advantages which were more than purely epistemic. As an operational centre bound to government (albeit being increasingly pushed into commercial independence), the Met Office was used to being told what to do, for example,

being told by the [Royal Air Force] what…they need to know over the next six hours to do one of their low altitude flights. So they were in some sense a little better than one might have suspected a NERC unit to be, in which they would have felt a little bit uneasy about being told what to do (interview, DoE science advisor).

In contrast to ‘a NERC unit’, akin to UGAMP, the Met Office could offer fast answers to political questions:

it looked like it was going to settle up to the fact that the UGAMP would go round…at the pace and rigour that academic community like to trundle along at. At least the Met Office would get something done. Because our feeling was that the pace of all the stuff politically was beginning to wind up a bit… [the Met Office] were naturally inclined to think they needed some guidance about what were *important* questions, as opposed to what were *interesting* questions (DoE science advisor, interview; emphasis added).

As correspondence went back and forth between the Met Office and DoE during 1989, the plans for the programme gradually took shape. The new centre would initially draw together 15 existing staff members, funded under the existing MoD contract, with 11 new employees funded by the new DoE contract. Emphasis was also placed on the centre having an open door to visiting scientists, in order to aid its functioning as a coordinating hub for UK climate change research. Yet despite this desired collaborative ethos, according to a senior UGAMP scientist (interview),

there was no consultation with those of us in academia. And in fact there was a feeling around that we were suddenly told this was the UK centre for climate research and climate modelling. So the spider had been put in there, and all we had in NERC was now seen as the web that fed to that spider.

Other established climate institutes also appeared a little perturbed when they learned of the plans. Tom Wigley of the University of East Anglia’s Climatic Research Unit (CRU) wrote to DoE in August of 1989: “I have heard rumours of such a centre being set up…Actually, a UK climate change centre already exists – it is called the Climatic Research Unit”.^14^ But Wigley’s unit was considered by DoE comparatively “small beer” when it came to modelling capacities next to the plans being drawn up with the Met Office.^15^ A well-timed telephone call from John Houghton assuaged the concerns of our above-quoted UGAMP scientist, although the latter declined Houghton’s suggestion of melding the new Centre with UGAMP. But for a time, it looked as though the Met Office centre might even outgrow the bounds of a *national* climate centre.

In early 1989, figures in the Met Office and DoE seemed jointly keen on the centre’s potential to be a focus for European climate modelling. One costed proposal from the Met Office included estimates both for the new Met Office centre and for a ‘European Centre for Climate Modelling’ to be located at Shinfield in Berkshire, near to the European Centre for Medium-range Weather Forecasts (ECMWF) which was opened in 1975. The annual cost of this European centre would peak at £9 million in 1992/3, including the costs of a new building. For David Fisk at DoE, this was clearly the intended direction, but perhaps on a larger scale than DoE had anticipated: “it’s a start, but it’s also ridiculous”, he noted on a copy of the proposal. Nonetheless, Houghton believed that “[e]nhancement of our effort during this period will also mean that the UK is in a strong position to play a leading part in the setting up (hopefully in the UK) of appropriate European or other international efforts”.^16^

But rather than two parallel centres, the dominant thinking began to focus on the new Met Office centre taking on a European role in the future, perhaps through a direct application for European funds. For Houghton, it was important to pre-empt any European designs and “to be seen to be active in order to try to prevent the EEC [European Economic Community] taking unwelcome initiatives”. Perhaps in response to the DoE’s balking at initial costings, Houghton emphasised the greater efficiency of collaboration between established or soon-to-be established European “centres of excellence”. But he foresaw “pressure” growing for a dedicated European centre, arguing that “we must be ready for it so that it can be landed in the UK”.^17^ Like Mason before him, Houghton was keen that the UK not be drowned-out by outside voices in climate modelling; if independence was to be sacrificed to Europe, the UK should at least be in the lead.^18^

But these European designs soon disappeared from correspondence and draft plans. The German state was clearly committed to its own climate scientists in Hamburg, investing funds into the DKRZ, while the emergence of the IPCC as a venue for international cooperation and coordination perhaps undermined the need for international centres: “elements of a cooperative world-wide venture already exist in the contacts between Met Office scientists and their European and US counterparts, fostered particularly by IPCC Working Group 1 activity”.^19^ Yet DoE was clearly still mindful of international competition: “Other groups currently have the potential to enter this area, including teams from Australia, Canada, the Federal Republic of Germany and France”, read the DoE input to a 1989 Science & Technology Select Committee enquiry into the Greenhouse Effect,^20^ although mentions of Japan and the USSR were struck-out of early drafts.^21^ In an internal DoE conversation about this “judicious reorientation of the science budget”, the European idea persisted. In a note following a DoE-Met Office meeting at which the idea was seemingly put to bed, Fisk speculated: “It will take some swallowing of pride for the brave BEF [presumably ‘British Expeditionary Force’] to go European, but I view it as inevitable…Most authorities agree in private that the final goal in 10–15 years’ time can only be met at the European level”.^22^ Senior DoE civil servant David Burr replied: “If we actually believe that we can’t continue to go it alone, should we be planning more consciously for alternatives?”^23^ Encapsulating what seemed to be a growing DoE anxiety that the Met Office, now focused entirely on its own national plans, might cede ground to other internationally ambitious institutions, David Warrilow, himself formerly of the Met Office, wondered in the margins of a draft proposal, “have we missed the boat?”

## The Final Goals of Climate Science (and Policy)

What was this ‘final goal’ mentioned in Fisk’s speculations? By the end of the 1980s climate modellers in both the US and the UK were seeking to go beyond equilibrium experiments with doubled CO_2_ levels to the pursuit of ‘transient’ simulations, which would model the response of the climate to changing atmospheric compositions, as they unfolded over time, with the help of a dynamic ocean model coupled to the atmosphere model. In addition to this greater temporal realism, DoE were keen on spatial realism; that is, on climate simulations with a fine spatial resolution, capable of resolving the climatic changes that might be expected on a national, rather than just global, scale. Fisk’s estimate of a 10–15 year wait for transient simulations with regional detail was optimistic in comparison with other estimates; DoE’s Select Committee input from 1989, for example, estimated 15–20 years for “regional ‘forecasts’”. An earlier ‘scenario’ of the development of UK climate research, circulated as an internal memo within DoE in early 1988, claimed “it is possible to speculate with some confidence how that picture [of future knowledge] will unfurl” (quoted in Shackley and Wynne 1996: 289). While quaintly over-ambitious in hindsight, the scenario’s claim that climate models could be “completed” within 25 years is particularly striking. Shackley and Wynne (1995, 1996) argue that this storyline of conquered uncertainty and resolved complexity performed an important social function within this emerging science-policy interface. As ‘boundary-ordering devices’ aiding in the complex management of uncertainty across science and politics, such schedules see the more “disturbing and challenging dimensions of uncertainty…translated into deterministically reducible ones”. These mutually-constructed visions of the future cement present relationships on the basis of what is to come. “The territory of the future is staked out and reclaimed for present commitments; hence the authority of the scientific program as currently formulated is neatly reinforced” (Shackley and Wynne 1996: 287).

### *Transience and Temporal Realism*

This informal ‘scenario’ of future climate research clearly informed the work schedule which was mutually constructed between the Met Office and DoE for the new climate centre. As drafts of the new contract evolved, transient and regional simulations moved up the list of prioritised ‘deliverables’. While scientists and research managers might view research schedules “with a mixture of irony and ritual”, they are important to policymakers, and often read “quite literally” (Shackley and Wynne 1996: 289). In this case, the pacing of the modelling requirements was shaped quite explicitly by anticipated developments in international policy. Hand-written notes by a DoE civil servant on the nature of the Met Office proposal stated that the requirement “is a definite product by 1992 before any convention on climate”, this definite product being a transient climate simulation. When the Met Office came back with a revised schedule showing a completed transient run in 1993, a DoE note in the margins queried – “1992?”^24^

DoE made it clear to the Met Office that expectations were high for a model which would be informing both Government and IPCC assessments:

I remember David Fisk saying right at the beginning about the model, ‘there are no prizes for coming second’. He was very certain that if the Hadley Centre model wasn’t the best, then we [DoE] shouldn’t use the Hadley Centre model, we should use another model (Met Office scientist, interview).

But the push for transience was not just motivated by an epistemic *telos* which defined progress and quality in climate modelling. Rather, for DoE, a transient simulation had a particular political potential:

in other areas we’d had a golden rule that you only ever really got environmental policy to move if either there were dead babies in the street or what you wanted to do was easier anyway. Golden rule. And unless you’ve got a transient model, you weren’t really picking up the dead babies… therefore the transient model had a lot more political clout in it, in handling your different people (DoE science advisor, interview).

Here, the potential of civil servants to persuade governments to act on an environmental problem is linked to the visibility of damage, destruction or, in this respondent’s gruesome metaphor, death. An equilibrium simulation, with its hypothetical response to an experimental doubling of atmospheric CO_2_, could not offer the kind of tractable realism demanded by this formulation of the link between persuasion and policy. Rather than just inviting a conversation about the end of the following century, with a transient experimental design a model could simulate, and policymakers could see, the steady or perhaps even catastrophic warming of the climate, and the accumulation of ‘impacts’, damages and losses along the way, the simulation “tracking them all”. Like earlier air pollution models (see above), a temporally realistic transient simulation could sit more comfortably with a regulatory culture founded on empiricism and high evidential standards.

By simulating climate change as a “cumulative” environmental problem – where effects are the result of past, current and future activities – the new temporality of transient simulations could also make a stronger link between schedules of future scientific advice, policy development, and climatic damage. If arguments for a strong policy response failed, “if we got rebutted the first time” (DoE science advisor, interview), climate change would continue happening, the warming and its side effects would continue to accumulate. If the first rebuttal concerned arguments based on abstract equilibrium experiments, then the content of these arguments would remain largely unchanged over time. Indeed, estimates of the equilibrium climate sensitivity to a doubling of CO_2_ have remained remarkably consistent between the late 1970s and today (IPCC 2014). To make the case again, one could only return to the same numbers. By contrast, a transient simulation of accumulating damage in ‘real time’ would have, for this DoE advisor, more persuasive “clout”, offering instead a more dynamic picture of a slowly unfolding future, seemingly inexorable in its computational logic, yet controllable insofar as policymakers could effect changes in the real world emissions profile: “If we’re into negotiations that were looking at different futures, that’s the thing that’s got to be modelled” (DoE official, interview). As in acid rain debates, the causal pathway became as important as the endpoint since the former is more responsive to policies than the latter.

In publications of the first transient run by the Hadley Centre (Murphy 1992; Murphy and Mitchell 1995), it is stressed that the simulated timescape of climate change should not be considered equivalent to calendar years. For example, it is explained how “the relative amplitude of the northern high latitude warming increases in the second half *of the experiment*”, rather than in the second half of the 21^st^ century (Murphy and Mitchell 1995: 78, emphasis added). Yet despite this careful policing of the boundary between experiment and the real world, it is clear that the temporal realism of transient simulations was a political attraction. The wish for a transient simulation before the 1992 international climate negotiations is a significant example of how the practices, priorities and norms of climate science were being ‘mutually constructed’ by scientific and political actors during this formative period (Shackley and Wynne 1995; Krueck and Borchers 1999). Policy demands were clearly setting the pace of scientific practice. Nonetheless, the eventual 1992 Rio commitment to stabilise emissions at 1990 levels by 2000 “was not determined by, or even closely ‘calibrated’ to, specific scientific knowledge yielded by current models” (ibid: 221) – not least because it was simply a ten-year commitment; the sort of medium-term timescale which climate models still struggle to handle (Meehl et al. 2014). The commitment was arguably more rooted in a precautionary mode of environmental policymaking, whereby the burden of proof of potential environmental harm is placed with opponents of regulatory action; if regulation is to be avoided, its opponents must prove the safety of whatever activity is seen as the source of harm. This stands in contrast to the preference for scientific consensus on the evidence for the possibility of harm as a prerequisite for regulation. As suggested above, a feature of UK regulatory culture and particularly of Conservative praxis has been the emphasis on high standards of scientific evidence. While the UK Government was busy putting together the Hadley Centre programme to establish a strong evidence base, other European nations were placing emphasis on the need for concrete policy targets:

the Dutch in particular were rather keen to do it the other way round really. Which is ‘let’s have some targets’. And then of course, you know, it’s the usual thing, ‘where is your energy policy if you’re a net fossil fuel exporter like UK?’ You can’t really see a reason why, on its own, you’d want 1990 levels at 2000 (DoE science advisor, interview).

For this senior DoE advisor, this emerging precautionary preference for picking “targets out of the air” stood in contrast to the coincident setting up of the Hadley Centre, which was “more deep, foundational in the way in which the Thatcher administration was thinking about how you did things…Get more science, [get more] knowledge”. Arguing the need for climate policy on the basis of a pioneering transient experiment would, this advisor suggested, accord with Conservative orientations to the future:

They don’t mind things that tell you *how* you’re going to economically grow, give them the shape and the colour of it, right? And if they started to sense this whole thing looked like, you know, *Limits to Growth* all over again, we’d be in real shit (ibid).

Rather than emphasising targets, and hence ‘limits’ to economic expansion, a transient run offered a legible and actionable future with the *telos* of economic growth, and the possibilities of conventional economic management, retained (see Russill 2016). The transient run was indeed completed in time for inclusion in an IPCC supplementary report prepared in advance of the Rio talks, although the Geophysical Fluid Dynamics Laboratory (GFDL) at Princeton had just beaten the Hadley Centre to the prize of performing the world’s first successful transient run (Manabe et al. 1991; IPCC 1992; Folland et al. 2004). The Hadley Centre run was completed despite the revelation in May 1989 that the Control Data Corporation was pulling support for the ETA-10 supercomputer, on which the Met Office had been relying as a replacement for its Cyber 205. This was “a bit of a disaster” for the carefully constructed work programme of both the new climate centre and the Office as a whole (interview, Met Office scientist). A Cray machine had to be sourced instead, adding more than £4 million in hardware and installation costs. But with the pressure on to come up with new results before 1992, the old Cyber supercomputer had to be kept running long after the opening of the Hadley Centre to perform the transient simulation, while the slow work of re-coding the GCM for the new Cray computer proceeded in parallel.

### *Regional Visions*

The prospect of regional climate simulation was another deliverable which rose up the Hadley Centre agenda during the 1989 negotiations with DoE. For the former DoE science advisor interviewed by us,

the regional modelling sort of crawled out of the IPCC process, because if you were going to tell China it was going to be barren and no rainfall and all the rest of it, there wasn’t any point talking about global average temperatures, in one way or another you had to say something about the South China Sea.

Regional climate modelling techniques had been emerging during the late 1980s at NCAR, where scientists had been contracted to perform simulations of future climates over potential nuclear waste storage sites (Dickinson et al. 1989). A developing ‘dynamical downscaling’ approach saw a meso-scale weather prediction model adjusted to the simulation of climate (mostly through the alteration of radiation schemes), and ‘embedded’ within a global climate model (e.g. Giorgi and Bates 1989). The global model would be run according to a given greenhouse gas emissions scenario. At the boundaries of the domain to be covered by the regional model, the global model would provide ‘boundary conditions’ (i.e. fluxes of matter and energy) for the regional simulation. As early work appeared to show, regional modelling offered the twin benefits of increased climatic realism as local topography could be accounted for, while increased physical resolutions accorded persuasively with “common sense notions of realism such as 3-D spatial meaning” (Shackley and Wynne 1995: 225-6).

To many at the time, the move towards regional climate simulation was seemingly “an entirely natural development” (Sir John Houghton, interview), a “reasonable” direction in which to extend modelling capacities (DoE official, interview). Regional models offered scientists the means of exploring regional responses to climate change, and even of re-connecting weather and climate through the dynamical simulation of local weather patterns – a task clearly at home at the Met Office, where weather and climate modellers shared computing facilities and often moved between weather and climate divisions (Sir John Houghton, interview). A regional model “was seen as essential” for refining the picture of future climate over the UK, which was represented very coarsely in global models (DoE official, interview). In 1990 the Climate Change Impacts Review Group was set-up under Martin Parry, with a mandate to assess the impact of future climate change on the UK. Although this body and its successor, the UK Climate Impacts Programme, did not make direct use of regional model output until the 2000s (Hulme and Dessai 2008), the pursuit of a more resolutely regional picture of the future of climate was underway by 1990. Regional climate prediction was a “policymaker’s delight” (UGAMP senior scientist, interview), a claim borne out by a DoE official (interview):

I’ll tell you why it was important to me and other people in the research programme. It was because of our experience with critical loads actually. And what matters to people is what’s going on here [gestures a small area].

*Interviewer*: how many salmon does it save?

*Respondent*: Yeah. And unless you can provide that sort of information, there’s no way that people can be confident about the impacts in their area.

Earlier civil service experience of the challenges of using models to persuade policymakers (e.g. with respect to acid rain) informed an enthusiasm for the spatial realism offered by regional climate modelling. Thatcher herself emphasised the work needed “to estimate the detailed distribution of the effects of global climate change…We want to know what’s happening in the regions” (Thatcher 1990). However, there was noticeable unease about the scheduling of such work in the Hadley Centre programme. At a meeting of the Meteorological Research Sub-Committee in April 1991, Sir John Mason “asked why the regional model featured so early in the plan”. The Hadley Centre’s David Bennetts emphasised the epistemic potential of the tool in helping to gain understanding of the behaviour of the global model, and to enable comparison with other techniques of generating regional information. However, perhaps detecting other pressures to develop and operationalise regional modelling, Brian Hoskins, Director of UGAMP at Reading, “urged caution on regional models”,^25^ based on concerns that unless global models could resolve meso-scale meteorological processes, downscaled predictions of regional change had questionable value. But despite these challenges, there was clear scientific and political momentum behind the Centre’s push for regional simulations.

### *The Hadley Centre on a Public Stage*

What’s in a name? Seemingly a lot about the politics of carving out a new institutional space in the borderlands of science and politics. As the Met Office and DoE together sought a suitable name for the new centre, a key criterion was a separate identity for the DoE-funded prediction work, as opposed to the “baseline MoD work on the subject”. The name should be non-parochial in character, yet flexible enough to allow “for possible future expansion if in the future the centre becomes an explicit focus of European research in this area”^26^ – the British Expeditionary Force may yet be crossing the Channel. Two DoE civil servants professed to having “invested an inordinate amount of time with very expensive Met Office senior officers over the name for the centre”.^27^ John Houghton “grudgingly” dropped his desire for the Meteorological Office to feature at the start of the official name, while George Hadley was eventually chosen over Harold Jeffreys as a target of tribute (interview, DoE official). The ‘Hadley Centre for Climate Research and Prediction’ was quickly abandoned for its unfortunate acronym. But Hadley himself was a hit – less obscure than previous suggestions, scientifically relevant for his 1730s work on the trade winds (perhaps an early ‘general circulation model’), and “ideal from the point of view of the Centre opening speech” – Hadley was a “barrister turned scientist”, and thus a mirror image of Thatcher, who was set to cut the ribbon. A Fellow of the Royal Society, Hadley was “British but with no obvious political disadvantages”^28^; indeed, as one DoE researcher sent to the archives reported, “There is much in the Hadley family that commends them, and especially George to honour: self-taught, skilled, industrious, a family that worked together”^29^; a model citizen of a Thatcherite Britain, perhaps.

DoE figures were clearly still concerned about other research groups taking umbrage at this centralisation of climate change work, and agonised that the name suggest “one centre among many”. As Alan Apling pointed out at the time, the branding of UGAMP “does not imply no one else does modelling”. But the key point for DoE was perhaps to obtain sufficient distinction from the rest of the Met Office, “so that DoE is unfettered in using the prediction programme politically and diplomatically”.^30^ The ‘Hadley Centre for Climate Prediction and Research’ thus emerged as a distinct and yet malleable political object, building on the predictive authority of the Met Office, yet something which could be used to bolster political arguments being made both within and by the Government.

With the work programme finalised (albeit subject to ongoing review) and a three-year rolling contract beginning at £5.7 million per year agreed,^31^ the Hadley Centre was officially opened on 25th May 1990. Thatcher, fresh from a briefing by John Houghton on the findings of the IPCC report which were being finalised that same week, penned a speech with Houghton’s assistance to deliver at the opening.^32^ The speech praised the work of the IPCC, argued that the climatic past was becoming a less reliable guide to the future, and emphasised the need for investment in modelling to “help us look into the future and predict more precisely the changes in our climate”. The address also included an announcement that “Britain is prepared to set itself the very demanding target of a reduction of up to 30 per cent in presently-projected levels of carbon dioxide emissions by the year 2005” – not so much a cut as a stabilisation at 1990 levels, falling short of the European target of returning to 1990 levels earlier, by 2000 (which would ultimately form the basis of the 1992 Rio commitment). Thatcher’s target was conditional on “others” being “ready to take their full share” (Thatcher 1990), thus wedding British policy to the UN process while furthering an awkward transatlantic brokerage between the northern Europeans and the US.^33^ In the US policy discourse had appeared increasingly obfuscatory, emphasising scientific uncertainties and eschewing talk of targets, to an even greater extent than in the UK. Thatcher’s announcement was greeted with disappointment by activists and the left-leaning press, who saw it as representing a “position that falls well short of virtually every call for action on global warming so far”^34^, including the suggested strategies of the IPCC report. For many commentators, Thatcher’s emphasis on ‘getting the science right’ looked increasingly like delaying tactics (Rose 1990).

## Discussion and Conclusions

What is remarkable about the scientific and political discussions of this period is how closely aligned they are. While the details of policy commitments may not have matched up to some of the demands being made by scientists and political advocates, a shared discourse of scientific complexity, of the powers of general circulation models and the promises of regional prediction can be detected in documents ranging from Met Office proposals through DoE memos to Prime Ministerial speeches.^35^ We can see in the emergence of the Hadley Centre the birth of the shared ‘policy culture’ described by Shackley and Wynne (1996), which placed complex climate models at the centre of new deliberations about energy policy and natural resource use, and eventually local planning (Hulme and Dessai 2008). Yet we want to suggest that this ‘policy culture’ was not an entirely new phenomenon – it had roots in a wider regulatory culture and was shaped by processes at various scales.

The emergence of the Hadley Centre reconfigured horizontal relationships between national climatological expertise and political authority. Climate scientists were brought into a new, close relationship with policymakers. This close relationship was not inevitable, as evidenced by its uniqueness (Shackley 2001; Krueck and Borchers 1999). Rather, it can be considered a historically contingent product of a regulatory culture which prizes ‘independent’, pragmatic judgement on issues which appear to transcend accepted visions of natural and social order. This desire for independent judgement was shared across science and politics: in the Met Office, senior scientists showed some scepticism towards the ‘alarmist’ claims of American modellers; in Government, civil servants managed to reconcile a potential “empiricist distrust” of models with emerging, more spatially and temporally ‘realistic’ forms of climate simulation (cf. Jasanoff 2011: 137). The capacity to predict was seen as allied to the capacity to adopt a political stance independent of both Europe and the US; empiricist caution was manifest here not in distrust of all models, but in a wish to develop a trusty model of one’s own. But this relationship between science and politics should not be conceived of linearly. Policy was not driven straightforwardly by science; the search for more certain science was in itself a policy choice.

It has often been claimed that Thatcher’s scientific background was crucial in her positive response to the claims of Houghton and others. We join Agar (2015) in refuting this argument. The Thatcher-the-scientist hypothesis cannot neatly explain her initial scepticism of the early 1980s, nor her apparent reluctance to commit to stringent policy measures in 1990 or her subsequent recourse to more sceptical arguments about the reality of climate change and the economic costs of action (see Thatcher 2002). As critical commentators at the time noted, Thatcher’s apparently Damascene conversion to the cause of climate change came with rhetoric reminiscent of the UK’s laggardly approach to acid rain and ozone depletion, where calls for more science could be interpreted as part of political delaying tactics (e.g. Rose 1990). While schedules of scientific research clearly helped scientists gain long-term government funding commitments (Shackley and Wynne 1996), they perhaps also contributed to the deflection of political action into the future. The promise of regional prediction, for example, accorded with Thatcher’s apparent wish “to know what is happening in the regions…we should have a better understanding of many of these things in ten or fifteen years’ time” (Thatcher 1990); such statements were interpreted by some critics at the time as ‘meaningful policy can therefore wait’.^36^ While an air of urgency emanated from both Met Office scientists and DoE officials, we might also argue that their emphasis on sound science – a key part of the rhetorical and practical toolkit of contemporary environmental policy – furnished ministers and other departments with a reason to ‘wait and see’, the public commitment to climate science offering “the appearance of activity while postponing substantive action”.^37^

The notion of civic epistemology can help us make sense of these apparent contradictions. As a “tried-and-true” repertoire of problem-solving (Jasanoff 2004: 40), a British civic epistemology of pragmatic empiricism, ‘sound science’ and independent judgement pervaded this institutionalisation of climate prediction, with diverse actors able to draw on this well-worn repertoire to build alliances and justify actions. There was thus something very British about the way the Hadley Centre was positioned as a new scientific institution. But this horizontal reorganisation of science and politics also occurred amid important vertical reorganisations. This was a period of a new ‘epistemic constitutionalism’ regarding climate change, whereby nation states “delegated the role of articulating and defending a shared epistemic foundation for global policy debates to a centralized, international institution”, that is, the IPCC (Miller 2009: 142). It is striking that the story of the Hadley Centre, from the first proposals to its opening, is bounded by the cycle of the IPCC First Assessment Report (FAR). The process of defining the Centre’s work programme proceeded alongside and, to some extent, in dialogue with the preparation of the FAR, which was part-supported by the British Government itself through the Met Office support unit which itself was located within the Hadley Centre by the time of the latter’s public opening, providing a “hotline” between the two institutions (Krueck and Borchers 1999: 116). Indeed, IPCC requirements continued to shape Hadley Centre research priorities over subsequent assessment cycles (ibid). This is a key example of the influence of the IPCC on the organisation and conduct of science on the national level – a feature not currently acknowledged by studies of the IPCC process, which is largely conceived as something passively responsive to pre-existing science, and only active in its delivery of a scientific message to policymakers (Hulme and Mahony 2010). We can see here how the IPCC was shaping scientific practice from the very start, in the shaping of institutions and the planning of work.

However, the shaping of the Hadley Centre illustrates how nation states were keen on exercising and strengthening their own epistemic sovereignty, a process still observable today (e.g. Mahony 2014). We therefore need to consider epistemic constitutionalism as a process occurring on multiple, interacting scales. Between 1988 and 1990 Thatcher took to both the national and international stage to profess a concern for climate change; a period when, the Cold War all but over, the global environment offered a new space for political internationalism. Reflecting on her choice to talk about the environment at the UN General Assembly in 1989, at which she announced the Hadley Centre plans, a former DoE advisor appealed not to her scientific background, but to her desire for political grandstanding at the ‘end of history’: “what else [were] you going to talk about?” (DoE science advisor, interview).

The emergence of the Hadley Centre thus symbolizes, and was part of, a key epistemic shift in understandings of climate and climatic change. More historical work is required to trace the impact of new practices of prediction – such as transient and regional – on these understandings. In a 1987 meeting between UK climate scientists organised by DoE and NERC, it was considered

crucial that the UK supports truly global and multi-disciplinary approaches to studying the climate system. Clearly, the potential local impacts of any suggested climate change are of paramount importance to the UK but we must guard against any suggestion that climate-change issues can in general be studied from a parochial regional viewpoint. Regional studies should be conducted with proper regard being paid to results stemming from a global approach.^38^

This ‘global approach’ was new, and was part of the co-production of newly global forms of knowledge and political order. It was an approach formed by the new hegemony of global climate models over alternative approaches to climate change which emphasised local environment-society interactions and determinants of risk (Russill 2016). This globalism was helped along by the vertical reorganisation of science and politics which saw institutions like the IPCC come to prominence amid political discourses of global action and cooperation (Miller 2004). We can read this warning against regional parochialism as concerning both the structures of scientific cooperation, and the emerging interest in regional climate prediction as enabled by global models. In this 1987 formulation, climate could no longer be considered a place-bound phenomenon (Heymann 2010). Instead, the global took precedence, both as an epistemic object and a political condition.

## Footnotes

1 Particularly the files AT 33/77 and AT 33/78 at the National Archives, Kew, UK. These were made public in February 2013.

2 Between January and July 2015 we interviewed three current or former employees of the Met Office, three from the Department of Environment, and one former head of a concurrent climate modelling programme. All had been active in negotiations between the Government and the Met Office during the period 1988–1992.

3 The Meteorological Office officially became the Met Office in 2000, but we use the latter title for brevity.

4 Shackley did not pass judgement on the merits of these different styles, and neither do we. While NCAR may appear a utopian bastion of intellectual autonomy, Shackley reported some envy among American modellers for the more rigid, task-focused structure of the Hadley Centre.

5 Houghton to Fisk, 15 Nov 1988, The National Archives [hereafter ‘TNA’] AT 33/77.

6 We might speculate too on the significance of the 1986 Chernobyl disaster in spurring new links between atmospheric modelling and governmental decision-making. The Met Office was operationally active during the incident, and was subsequently sanctioned by government to generate new emergency response modelling capabilities. A new atmospheric dispersion model, developed specifically for nuclear accidents, was operational by 1988 (Jones et al. 2007).

7 Mason, J., ‘Record of meeting to discuss UK research on climate change’, TNA AT 33/77, p 1.

8 Ibid, pp 1–2. Mason perhaps had in mind the UK’s hosting of the European Centre for Medium-range Weather Forecasts (ECMWF), opened in 1975. As one senior UGAMP (Universities Global Atmospheric Modelling Project) scientist suggested, “John Mason wanted it [ECMWF] here, partly to keep his eye on it I think” (interview).

9 Martin-Nielsen (2015) describes tensions between the computationally-minded Mason and Hubert H. Lamb’s more interdisciplinary approach to understanding climate. This clash of cultures culminated in Lamb’s founding of the Climatic Research Unit (CRU) at the University of East Anglia.

10 Mason, Record of meeting, p 2.

11 Houghton to Fisk, 15 Nov 1988, TNA AT 33/77.

12 White, ‘UK National Climate Modelling Research Centre: A strategy document’, TNA AT 33/77.

13 Apling to Fisk, n.d., 1989, TNA AT 33/77.

14 Wigley to Warrilow, 22 Aug 1989. TNA AT 33/77.

15 Annotated note, Warrilow to Apling, on Wigley to Warrilow, 22 Aug 1989. In the late 1980s, CRU scientists developed a simple one-dimensional climate model called STUGE (which later evolved into MAGICC) which subsequently proved influential in policy assessments, but it required parameter inputs such as climate sensitivity estimates from GCMs and was calibrated against them.

16 Houghton to Fisk, 3 March 1989. TNA AT 33/77.

17 Houghton to Fisk, 6 March 1989. TNA AT 33/77.

18 Houghton’s wish to ‘land’ any European centre echoes the earlier arrival of ECMWF in the UK (see note 8). Landing another European atmospheric science institution in the UK so soon would have been quite a diplomatic achievement.

19 Burr to Fisk, 31 May 89. TNA AT 33/77.

20 See House of Lords Select Committee on Science and Technology, Session 1988–89, 6th Report, Greenhouse Effect, October 1989.

21 TNA AT 33/77.

22 Fisk to Burr, 6 July 1989. TNA AT 33/77.

23 Burr to Fisk, 11 July 1989. TNA AT 33/77.

24 Draft Schedule, TNA AT 33/77.

25 Minutes of the 22nd Meeting of the Meteorological Research Sub-Committee, 15 April 1991. Document kindly supplied by David Carson.

26 Draft submission from Dr Fisk to PS-SOS on the climate prediction initiative, TNA AT 33/78.

27 Apling to Fisk, 26 Jan 1990. TNA AT 33/78.

28 Draft submission from Dr Fisk to PS-SOS on the climate prediction initiative, TNA AT 33/78.

29 Goodier to Apling, 22 March 1990. TNA AT 33/78.

30 Apling to Fisk, 26 Jan 1990. TNA AT 33/78.

31 Between £12m and £16m at present values.

32 This high-profile event recalls the Met Office’s first ever press conference, convened in November 1965 by John Mason to announce the start of operational NWP (Hall 2015b). Interesting questions are raised here concerning the links between computer modelling and the public performance of scientific authority.

33 Nicholson-Lord, D., ‘Lord make us green, but not yet’, *The Independent*, 27 May 1990.

34 Ibid.

35 In the latter case, of course, we have the example of one speech co-written by a Prime Minister and the chief of a national meteorological service.

36 See, for example, Nicholson-Lord, D., ‘Lord make us green, but not yet’, *The Independent*, 27 May 1990.

37 This quote is from a Department of Energy civil servant and is contained in the leaked minutes of a meeting on climate policy between the Confederation of British Industry and the Department of Energy. See Rose (1990: 294).

38 Rapporteurs’ draft notes, ‘Man-made Climate Change: Planning the UK Research Strategy’, Institute of Hydrology, Wallingford, 28–29 April 1987. Provided to the authors by David Carson.
